# The *Ralstonia solanacearum* Type III Effector RipAY Is Phosphorylated in Plant Cells to Modulate Its Enzymatic Activity

**DOI:** 10.3389/fpls.2017.01899

**Published:** 2017-11-07

**Authors:** Yali Wei, Yuying Sang, Alberto P. Macho

**Affiliations:** ^1^Shanghai Center for Plant Stress Biology, CAS Center for Excellence in Molecular Plant Sciences, Shanghai Institutes of Biological Sciences, Chinese Academy of Sciences, Shanghai, China; ^2^University of Chinese Academy of Sciences, Beijing, China

**Keywords:** effector phosphorylation, effector activation, post-translational modification, plant immunity, plant disease

## Abstract

Most bacterial pathogens subvert plant cellular functions using effector proteins delivered inside plant cells. In the plant pathogen *Ralstonia solanacearum*, several of these effectors contain domains with predicted enzymatic activities, including acetyltransferases, phosphatases, and proteases, among others. How these enzymatic activities get activated inside plant cells, but not in the bacterial cell, remains unknown in most cases. In this work, we found that the *R. solanacearum* effector RipAY is phosphorylated in plant cells. One phosphorylated serine residue, S131, is required for the reported gamma-glutamyl cyclotransferase activity of RipAY, responsible for the degradation of gamma-glutamyl compounds (such as glutathione) inside host cells. Accordingly, non-phosphorylable mutants in S131 abolish RipAY-mediated degradation of glutathione in plant cells and the subsequent suppression of plant immune responses. In this article, we examine our results in relation to the recent reports on the biochemical activities of RipAY, and discuss the potential implications of phosphorylation in plant cells as a mechanism to modulate the enzymatic activity of RipAY.

## Introduction

Many bacterial plant pathogens secrete dozens of proteins inside plant cells using a type-III secretion system (T3SS). These proteins (type III-secreted effectors; T3Es) are key determinants of the interaction between pathogen and host, contributing to the alteration of plant cellular processes to promote infection (Deslandes and Rivas, 2012; Macho, 2016). T3E activities can also be perceived by resistant plants, leading to the activation of immune responses (Khan et al., 2016). The bacterial pathogen *Ralstonia solanacearum* causes the devastating bacterial wilt disease in numerous hosts, constituting an important threat to agriculture (Mansfield et al., 2012), but is also an emerging system for the study of T3Es (Deslandes and Genin, 2014). Strains from *R. solanacearum* express more than 70 different effectors (Peeters et al., 2013), and the T3S-dependent secretion or translocation of most of them has been experimentally validated (Cunnac et al., 2004; Mukaihara et al., 2010). Remarkably, many of these effectors contain domains with predicted enzymatic activities, including acetyltransferases, phosphatases, and proteases, among others (Dean, 2011; Peeters et al., 2013). After delivery inside plant cells, these proteins have the potential to associate with plant molecules in a specific or unspecific manner, catalyzing their modification to promote bacterial proliferation and the development of disease (Dean, 2011; Macho and Zipfel, 2015). This raises the intriguing question of how the enzymatic activities of these bacterial effectors are maintained in an inactive state in the bacterial cell, but become active inside host cells after their delivery through the T3SS. High degree of substrate specificity could be a potential mechanism employed by bacterial enzymes to prevent activity toward bacterial substrates. However, strict substrate specificity may be difficult to achieve in many cases, or impossible in scenarios where the substrate molecule is also present in bacterial cells. Specific modification of T3Es inside eukaryotic cells has been proposed as a mechanism to explain the selective activation of T3E enzymatic activities after their delivery into host cells. Accordingly, in recent years, several T3Es from bacterial pathogens have been shown to undergo activation through interaction with host factors or post-translational modifications (PTMs) inside plant cells, such as phosphorylation, ubiquitination, or lipidations (Popa et al., 2016).

The *R. solanacearum* T3E RipAY is present in all the *R. solanacearum* strains sequenced to date (Peeters et al., 2013), and contains a ChaC domain, exhibiting gamma-glutamyl cyclotransferase (GGCT) activity (Fujiwara et al., 2016; Mukaihara et al., 2016). RipAY employs this GGCT activity to degrade the redox buffer glutathione (and potentially other gamma-glutamyl compounds) in plant cells, causing a suppression of plant immune responses (Fujiwara et al., 2016; Mukaihara et al., 2016; Sang et al., 2016). Interestingly, although the degradation of plant glutathione seems a powerful strategy for bacterial pathogens to subvert plant physiological functions, no other T3E has been reported to exhibit GGCT activity. A potential danger caused by the bacterial production of a GGCT enzyme is the degradation of bacterial gamma-glutamyl compounds, and therefore GGCT activity must be selectively activated after delivery inside host cells. Eukaryotic thioredoxins have been shown to contribute to the activation of RipAY GGCT activity *in vitro*, which is otherwise not detectable after RipAY purification from bacterial cells (Fujiwara et al., 2016; Mukaihara et al., 2016). However, the mechanism of thioredoxin-mediated activation of RipAY remains unknown. In this work, we report that RipAY is phosphorylated in plant cells. Site-directed mutagenesis of the phosphorylated residues indicates that a serine residue is required for the RipAY-mediated degradation of glutathione and suppression of immune responses triggered by bacterial elicitors, although it is not required for the reported interaction with plant thioredoxins. We discuss the possible interpretations of this result, raising questions and offering new perspectives for the study of phosphorylation in plant cells as a potential mechanism to modulate enzymatic activities of RipAY and other *R. solanacearum* T3Es.

## Materials and Methods

### Plant Material, Growth Conditions, and Transient Expression Assays

*Nicotiana benthamiana* plants used in this study were grown in an environmentally controlled growth room at 22°C with a 16 h photoperiod. *Agrobacterium*-mediated transient expression was performed as described (Li, 2011). Bacterial suspensions were adjusted to a final OD^600^ of 0.5 before infiltration.

### Chemicals

The flg22 peptide (sequence from *Pseudomonas syringae* pv. *tomato* DC3000; TRLSSGLKINSAKDDAAGLQIA) was purchased from Abclonal, United States. Sequencing-grade modified trypsin was purchased from Promega (Madison, WI, United States). The Nanosep centrifugal filter units with Omega membrane (MWCO 10 kDa) were bought from Pall Corporation (New York, NY, United States). All other chemicals were purchased from Sigma-Aldrich (St. Louis, MO, United States) unless otherwise stated.

### Immunoprecipitation and LC-MS/MS Analysis

Immunoprecipitation assays and Western blots were performed as described previously (Sang et al., 2016). Briefly, total *N. benthamiana* proteins were extracted and incubated with GFP-trap beads (ChromoTek, Germany). After washing, IPed proteins were stripped from the beads and separated on precast SDS-PAGE gels (Bio-Rad, Hercules, CA, United States). Western blot was performed using the anti-FLAG (Sigma) and anti-GFP (Abiocode, Agoura Hills, CA, United States) primary antibodies, respectively. Blots were stained with Coomassie Brilliant Blue to verify equal loading. Protein digestion, liquid-chromatography coupled to tandem mass-spectrometric analysis and peptide identification were performed as described (Sang et al., 2016).

### ROS and Glutathione Measurements

The measurement of the flg22-triggered ROS burst is detailed in Sang and Macho (2017). Total cellular glutathione was measured using the Glutathione Assay Kit (Sigma-Aldrich) according to the manufacturer’s instructions, as described (Sang et al., 2016).

### Constructs and Site-Directed Mutagenesis

Constructs for transient expression of RipAY and thioredoxins have been described before (Sang et al., 2016). New RipAY mutant variants were generated as described previously (Sang et al., 2016), using the primers listed in Supplementary Table S1.

### Confocal Microscopy and Image Processing

Leaves from 4- to 5-week-old *N. benthamiana* plants were infiltrated with *Agrobacterium* to induce the expression of RipAY-GFP or the indicated mutant versions. Two days after inoculation, RipAY-GFP subcelullar localization was observed in pavement epidermal cells. GFP imaging was performed on an inverted laser scanning confocal microscope (Leica TCS SP8) equipped with LEICA LAS X image acquisition software. The green fluorescent protein (GFP) was excited by a 488 nm argon laser at a strength of 20% and detected at 501–528 nm (Em).

## Results

### RipAY Is Phosphorylated in Plant Cells

**FIGURE 1:**
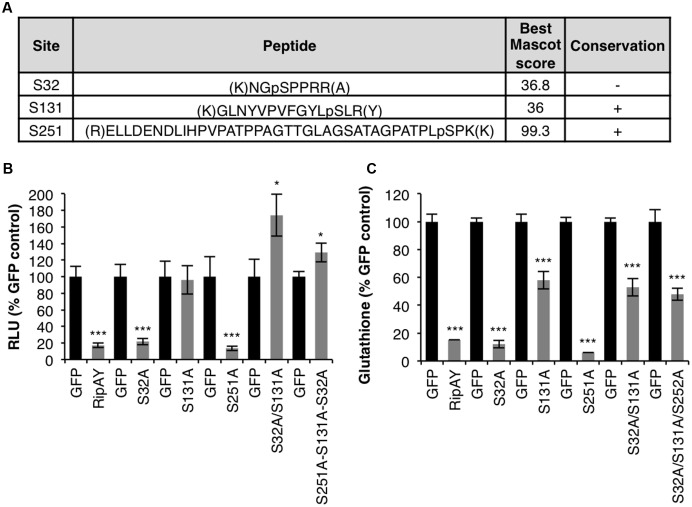
Relevance of phosphorylation for RipAY activity in plant cells. **(A)** Tryptic peptides containing phosphorylated serine or threonine residues found after IP+LC-MS/MS of RipAY-GFP expressed in *N. benthamiana*. Only peptides with a Mascot ion score above 35 were considered. Lowercase p indicates the phosphorylation site. The conservation of the phosphorylated sites in RipAY sequences from *R. solanacearum* strains from different phylotypes is indicated (see **Supplementary Figure S2** for details). **(B)** *A. tumefaciens* was used to induce the transient expression of RipAY-GFP (or the indicated mutant variants) in half of a *N. benthamiana* leaf and GFP in the other half. The graph shows total oxidative burst triggered by 100 nM flg22 in *N. benthamiana* tissues 2.5 days post-inoculation (dpi) with *A. tumefaciens*, and measured in a luminol-based assay as relative luminescence units (RLUs). Values are average ± SE (*n* = 8). **(C)** Total glutathione content in *N. benthamiana* tissues 2.5 dpi with *A. tumefaciens* inducing the expression of GFP, RipAY-GFP (or the indicated mutant variants). Values are average ± SE (*n* = 3). Asterisks indicate significant differences compared to the corresponding GFP control (^∗∗∗^*P* < 0.001; ^∗^*P* < 0.05). The experiments were repeated three times with similar results.

In order to identify RipAY residues post-translationally modified in plant cells, we previously performed *Agrobacterium*-mediated transient expression of RipAY (cloned from the GMI1000 strain; phylotype 1) fused to a green-fluorescent protein tag (RipAY-GFP) in *Nicotiana benthamiana* cells (Sang et al., 2016). GFP immunoprecipitation (IP) followed by liquid chromatography and tandem-mass spectrometry (LC-MS/MS) allowed us to identify several RipAY peptides containing phosphorylated serine (S) and threonine (T) residues (**Figure 1A** and **Supplementary Figure S1**). Among these residues, S131 and S251 are located within the ChaC domain (**Supplementary Figure S1A**), and are conserved in RipAY sequences from several *R. solanacearum* strains representative from different phylotypes (**Figure 1A** and **Supplementary Figure S2**). An additional phosphorylated residue, S32, was only conserved in strains from the phylotype I, although RipAY sequences from other strains present serine residues in the position 30 or 31 (**Supplementary Figure S2**).

### S131 Is Important for RipAY Activity *in Planta*

RipAY-GFP transiently expressed in *N. benthamiana* is able to suppress the rapid generation of reactive oxygen species (ROS) after treatment of plant tissues with the immune elicitor flg22 (Sang et al., 2016). In order to determine whether the potential phosphorylated residues found in the LC-MS/MS assays are important for RipAY immune suppression activity, we performed single or multiple site-directed mutagenesis of these residues, substituting them to the non-phosphorylable residue alanine (A). As shown in the **Figure 1B**, the S131A mutation abolished RipAY ability to suppress flg22-triggered ROS burst (**Figure 1B**). Strikingly, although the single S32A mutation did not affect RipAY suppression of flg22-triggered ROS, expression of the double S32A/S131A mutant consistently increased this response in comparison with GFP-expressing control tissues (**Figure 1B**).

It has been previously shown that the ability of RipAY to suppress immune responses is associated to the degradation of plant glutathione through its GGCT activity (Mukaihara et al., 2016; Sang et al., 2016). To test whether the potential phosphorylated residues are important for RipAY catalytic activity, we measured glutathione content in plant tissues expressing RipAY or the different mutants carrying the mentioned amino-acid substitutions. *N. benthamiana* tissues expressing RipAY showed a dramatic reduction of glutathione content (10–15%) compared with GFP-expressing control tissues (**Figure 1C**). Mutants containing the S131A mutation were partially impaired in glutathione degradation in comparison with wild-type RipAY (50–60% compared to control tissues; **Figure 1C**). Besides S131A, additional mutations in other phosphorylated residues did not significantly affect RipAY ability to degrade plant glutathione (**Figure 1C**).

We next tested whether S131 phosphorylation could contribute to the activation of RipAY function. Mutation of serine to aspartic acid (D) sometimes resembles the addition of a phosphate group on a serine residue, mimicking a phosphorylation event. However, a S131D mutation abolished RipAY ability to suppress flg22-triggered ROS burst (**Supplementary Figure S3**).

The mutagenesis of the phosphorylated residues did not affect RipAY accumulation (**Supplementary Figure S4A**). As we reported before, we consistently detected a double band corresponding to RipAY in Western blot assays (Sang et al., 2016; **Supplementary Figure S4**), which may be caused by PTMs of the protein. This double band became more obvious when using acrylamide gels containing Phos-Tag, which binds phosphorylated residues, slowing down the migration of phosphorylated proteins (Kinoshita et al., 2006; **Supplementary Figure S4B**), suggesting that this band-shift may be caused by phosphorylation. Interestingly, the S32A mutation altered the ratio between these bands, increasing the concentration of the fast-migrating band (**Supplementary Figure S4**), suggesting that phosphorylation in S32 accounts for most RipAY phosphorylation, or that phosphorylation of this site is required for the subsequent phosphorylation of others.

### The S32A and S131A Mutations Do Not Affect RipAY Subcellular Localization or Interaction with Plant Thioredoxins

**FIGURE 2:**
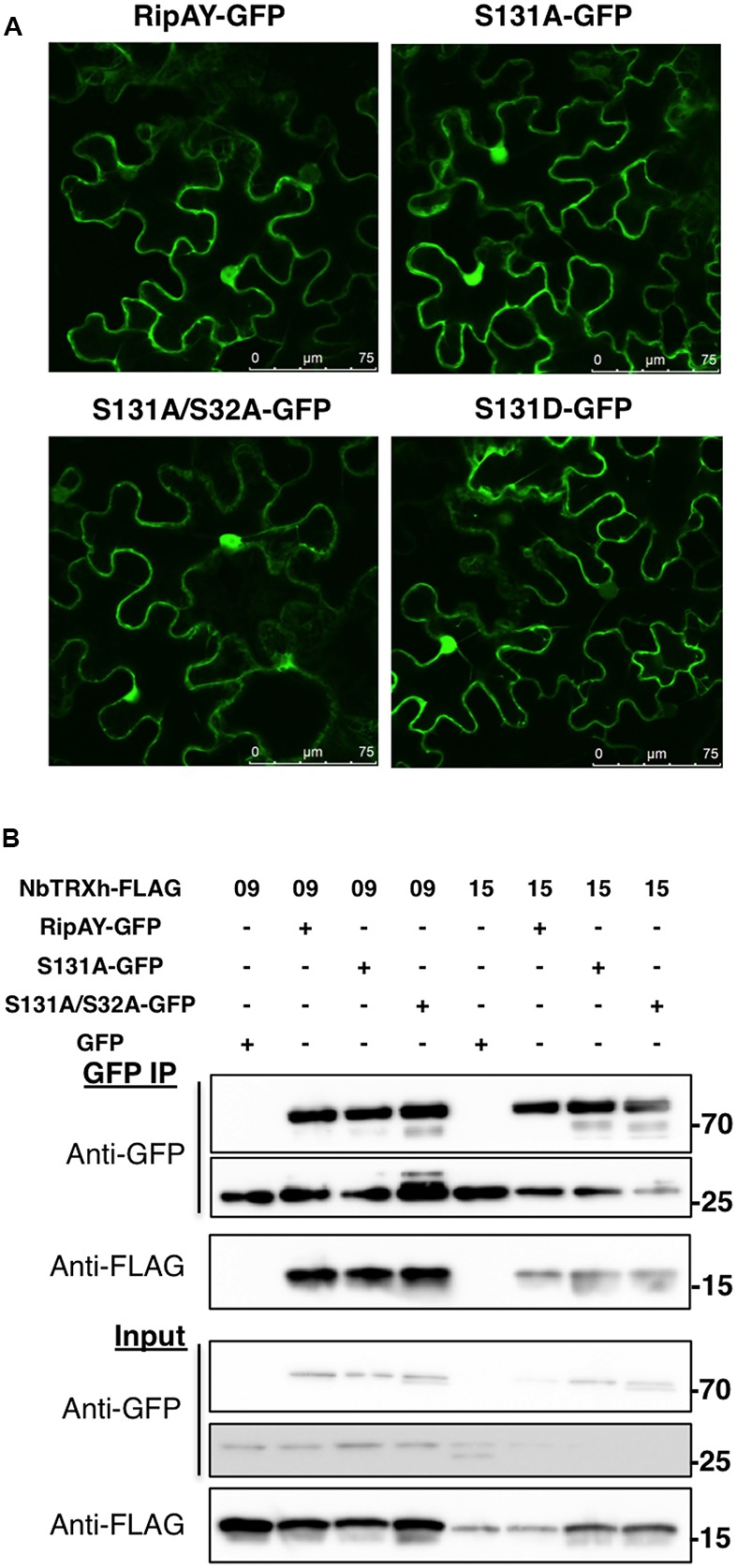
The S32A and S131A mutations do not affect RipAY subcellular localization or interaction with plant thioredoxins. **(A)** Representative images of RipAY-GFP and the indicated mutant variants transiently expressed in *N. benthamiana* leaf pavement cells. Size bars (75 μm) are shown for reference. **(B)** GFP or RipAY-GFP were co-expressed with the indicated h-type thioredoxins in *N. benthamiana* before immunoprecipitation using GFP-trap beads. Immunoblots were analyzed using anti-GFP or anti-FLAG antibody. Molecular weight (kDa) marker bands are indicated for reference. A band corresponding to approximately 27 kDa can be detected in RipAY samples, and this most likely reflects the cleavage of the GFP tag in the process of protein overexpression and extraction. The experiments were repeated twice with similar results.

Protein phosphorylation can affect protein-protein interactions and the subcellular localization of phosphorylated proteins. We previously reported that RipAY-GFP localizes at the cytoplasm and nucleus of *N. benthamiana* cells, and associates with several thioredoxins (TRXs) *in planta* (Sang et al., 2016). Association with plant TRXs has been shown to contribute to RipAY activation *in vitro* (Fujiwara et al., 2016; Mukaihara et al., 2016). Since the S32A mutation altered RipAY band-shift and S131A abolished RipAY activity, we tested whether these mutations affect the subcellular localization of RipAY-GFP or its association with plant thioredoxins. Confocal microscopy showed that transiently expressed RipAY-GFP localizes at the cytoplasm and nucleus of *N. benthamiana* cells, and the S131A or S32AS131A mutations did not affect this subcellular localization (**Figure 2A**). Similarly, none of these mutations altered the reported RipAY-GFP association with NbTRX09 and NbTRX15 in Co-IP assays (**Figure 2B**).

## Discussion and Perspectives

Upon transient expression in *N. benthamiana* cells, we were able to detect phosphorylation in several RipAY peptides (**Figure 1A**). Mutagenesis of the phosphorylated residues to alanine indicated that S32A alters the putative phosphorylation-associated bandshift of RipAY, and mutations in S131 abolish its function. Interestingly, although S32 seems to be the major site responsible for the RipAY bandshift (**Supplementary Figure S4B**), mutations in that residue did not affect the RipAY-mediated degradation of glutathione or suppression of immune-related ROS burst (**Figure 1**). Therefore, it remains unclear whether phosphorylation in S32 (and the potential subsequent phosphorylation of other sites) is not relevant for RipAY activity or required for other activities not measured in our assays. Moreover, although the S131A mutation completely abolished the ability of RipAY to suppress the immune-related ROS burst, it only partially compromised its ability to degrade glutathione (**Figure 1C**), indicating that S131 has a quantitative contribution, but is not fully required, for the GGCT activity of RipAY.

We failed to induce activation of RipAY *in planta* by substituting S131 to an aspartic acid residue. It is still possible that phosphorylation of S131 is important for activity, but S131D cannot mimic phosphorylation. Of note, phosphorylation is a dynamic process, and the addition/removal of a phosphate group may need to be regulated in spatiotemporal manner. In such situations, the substitutions to negatively charged amino acids might not mimic phosphorylation, but rather result in loss of function, as it has been observed for phospho-mimic mutants before (Oh et al., 2009).

Importantly, S131 has been reported to be present within the putative substrate-binding site of the RipAY ChaC domain (Fujiwara et al., 2016). A S131A mutation partially suppressed RipAY ability to degrade glutathione *in vitro* and abolished RipAY inhibition of yeast growth (Fujiwara et al., 2016). It is possible that both phospho-dead and phospho-mimic mutations disrupt the RipAY ChaC substrate-binding site and thus disrupt activity. This raises the possibility that the observed *in planta* phosphorylation of S131 is not biologically relevant, although we should not rule out that plant kinases may be actively phosphorylating this site in an attempt to disrupt RipAY activity. This has been shown for Pto kinase, which abolishes AvrPtoB E3 ubiquitin ligase activity by phosphorylating an essential threonine residue in the catalytic domain of AvrPtoB (Ntoukakis et al., 2009). In that case, also, both phospho-dead and phospho-mimic mutations in the phosphorylated residue (S450A or S450D, respectively) abolish the catalytic E3 ligase of the effector (Ntoukakis et al., 2009). In the case of transiently expressed wild-type RipAY in *N. benthamiana* cells, we can find peptides phosphorylated in S131, but this transient expression is still able to cause the degradation of glutathione and suppression of immune responses. Therefore, either this mechanism of plant-mediated RipAY inactivation is not predominant in natural conditions, or a portion of RipAY may be inactivated by phosphorylation, but effector overexpression in our assays may allow enough active RipAY to degrade glutathione. The PTM of important residues in the active sites of T3Es could constitute a powerful strategy for plants to inactivate their catalytic activities. Being highly conserved sites subjected to strong positive selection (due to their importance for the catalytic activity), it would be difficult for the pathogen to alter their sequence in order to avoid inactivation in resistant hosts.

The presence of TRXs has been shown to activate RipAY GGCT activity *in vitro*, although the underlying mechanism is still unknown (Fujiwara et al., 2016; Mukaihara et al., 2016). Therefore, we sought to determine whether S131 phosphorylation would be required for binding to TRXs as a mechanism to contribute to RipAY activation. However, RipAY S32A/S131A still associated with TRXs *in planta* (**Figure 2**), suggesting that phosphorylation of these sites is not required for TRX binding. Interestingly, although TRXs catalyze the reduction of disulphide bonds of target proteins, a mutation in the sole cysteine (C) residue of RipAY (C333S) did not abolish TRX binding or any of RipAY activities *in vivo* and *in vitro* (Fujiwara et al., 2016; Sang et al., 2016). Recombinant RipAY purified from *Escherichia coli* cells shows thioredoxin-dependent GGCT activity *in vitro* (Fujiwara et al., 2016; Mukaihara et al., 2016). This suggests that RipAY phosphorylation by plant kinases is not required for GGCT activity *in vitro*. However, we have often observed several phosphorylated peptides in recombinant effector proteins purified from *E. coli* in conditions of overexpression, and this is a common phenomenon observed during the purification of active recombinant kinases from *E. coli* cells. Therefore, in conditions of overexpression, effectors can be phosphorylated by *E. coli* kinases and thus be purified in phosphorylated form, enabling *in vitro* reactions that require the phosphorylation of effector residues. Alternatively, it is possible that RipAY phosphorylation modulates the interaction of RipAY with substrates or other host factors required for its activity in plant cells, but dispensable for *in vitro* assays toward generic substrates. Structural biology approaches will be key to determine the potential relevance of the phosphorylated residues and the interaction with host factors for the activation of the catalytic activity of RipAY.

Phosphorylation has been found in an increasing number of T3Es from different plant pathogens (Popa et al., 2016), such as the *Pseudomonas syringae* T3E HopQ1, which contains a domain with predicted ADP-ribosyltransferase activity. HopQ1 is phosphorylated in plant cells, and this is required for its interaction with 14-3-3 proteins, which determine its subcellular localization and molecular activity (Giska et al., 2013; Li et al., 2013). *R. solanacearum* contains a HopQ1 ortholog, named RipB, which has conserved 14-3-3-binding sites and associates with 14-3-3 proteins in plant cells (Giska et al., 2013). Additionally, we have found phosphorylated peptides in several other *R. solanacearum* T3Es with predicted enzymatic activities after transient expression in plant tissues followed by protein extraction, co-IP and LC-MS/MS analysis (data not shown). These findings suggest that phosphorylation may be an extended mechanism for the modulation of the catalytic activity of *R. solanacearum* T3Es in plant cells, although the validation of this hypothesis will require the specific biochemical characterization of each T3E independently. Furthermore, the development of more sensitive proteomic approaches will help determining whether these phosphorylation events take place in natural conditions during the infection and whether other T3Es injected simultaneously inside plant cells may play a role in these modifications.

Using the RipAY amino acid sequence, a search in the Scansite3 server^1^ predicted that S32 and S251 are phosphorylated by kinases from the proline-dependent serine/threonine kinase group, such as cyclin-dependent kinases. However, we were not able to detect such kinases among RipAY interactors in our IP+LC-MS/MS approach *in planta* (Sang et al., 2016). To date, only two plant kinases, Pto and PIK1, have been found to phosphorylate T3Es (AvrPtoB and AvrBsT, respectively; Ntoukakis et al., 2009; Kim et al., 2014). The identification of plant kinases involved in the phosphorylation of *R. solanacearum* T3Es will be essential to fully understand their biochemical activation, and will be an important step toward the biotechnological application of this fundamental knowledge to the development of disease-resistant plants. Given the transient nature of kinase-substrate interactions, this constitutes a challenge for future studies.

## Author Contributions

YW, YS, and AM designed experiments, performed experiments, and analyzed data. AM wrote the paper.

## Conflict of Interest Statement

The authors declare that the research was conducted in the absence of any commercial or financial relationships that could be construed as a potential conflict of interest.

## Footnotes

1 http://scansite3.mit.edu/

## Supplementary material

FIGURE S1 RipAY is phosphorylated in several serine residues. **(A)** Diagram of RipAY indicating the ChaC domain (responsible for the GGCT activity) in red and the observed phosphorylation sites. E216 indicates the catalytic glutamic acid residue essential for GGCT activity. **(B)** Representative mass spectra of the observed phosphorylated peptides indicated in the **Figure 1A**.

FIGURE S2 Amino acid sequence alignment showing the conservation of the phosphorylated residues among RipAY from different *R. solanacearum* strains. The strains are representative from the different *R. solanacearum* phylotypes: phylotype 1: FJAT91, SD54, YC45, FQY4, GMI1000; phylotype 2: UW551 and Molk2; phylotype 3: CMR15; phylotype 4: Psi07. The phosphorylated residues identified in this work in RipAY from GMI1000 are marked with an asterisk

FIGURE S3 The S131D mutation abolishes RipAY suppression of flg22-triggered ROS. *A. tumefaciens* was used to induce the transient expression of RipAY-GFP (or the S131D mutant) in half of a *N. benthamiana* leaf and GFP in the other half. The graph shows total oxidative burst triggered by 100 nM flg22 in *N. benthamiana* tissues 2.5 days post-inoculation (dpi) with *A. tumefaciens*, and measured in a luminol-based assay as relative luminescence units (RLUs). Values are average ± SE (*n* = 8). Asterisks indicate significant differences compared to the corresponding GFP control at *P* < 0.001. The experiments were repeated at least three times with similar results.

FIGURE S4 All the RipAY mutant variants accumulate to wild-type levels. **(A)** Western blot assay showing the accumulation of the mutant variants shown in the **Figures 1**, **2** and **Supplementary Figure S3**. **(B)** Western blot assay using a Phos-Tag PAGE gel. Immunoblots were analyzed using anti-GFP antibody. Molecular weight (kDa) marker bands are indicated for reference.

## References

[B1] Cunnac S. Boucher C. Genin S. (2004). Characterization of the *cis*-acting regulatory element controlling HrpB-mediated activation of the type III secretion system and effector genes in *Ralstonia solanacearum*. *J. Bacteriol.* 186 2309–2318. 15060033 10.1128/JB.186.8.2309-2318.2004 PMC412162

[B2] Dean P. (2011). Functional domains and motifs of bacterial type III effector proteins and their roles in infection. *FEMS Microbiol. Rev.* 35 1100–1125. 10.1111/j.1574-6976.2011.00271.x 21517912

[B3] Deslandes L. Genin S. (2014). Opening the *Ralstonia solanacearum* type III effector tool box: insights into host cell subversion mechanisms. *Curr. Opin. Plant Biol.* 20 110–117. 10.1016/j.pbi.2014.05.002 24880553

[B4] Deslandes L. Rivas S. (2012). Catch me if you can: bacterial effectors and plant targets. *Trends Plant Sci.* 17 644–655. 10.1016/j.tplants.2012.06.011 22796464

[B5] Fujiwara S. Kawazoe T. Ohnishi K. Kitagawa T. Popa C. Valls M. (2016). RipAY, a plant pathogen effector protein, exhibits robust gamma-glutamyl cyclotransferase activity when stimulated by eukaryotic thioredoxins. *J. Biol. Chem.* 291 6813–6830. 10.1074/jbc.M115.678953 26823466 PMC4807269

[B6] Giska F. Lichocka M. Piechocki M. Dadlez M. Schmelzer E. Hennig J. (2013). Phosphorylation of HopQ1, a type III effector from *Pseudomonas syringae*, creates a binding site for host 14-3-3 proteins. *Plant Physiol.* 161 2049–2061. 10.1104/pp.112.209023 23396834 PMC3613475

[B7] Khan M. Subramaniam R. Desveaux D. (2016). Of guards, decoys, baits and traps: pathogen perception in plants by type III effector sensors. *Curr. Opin. Microbiol.* 29 49–55. 10.1016/j.mib.2015.10.006 26599514

[B8] Kim N. H. Kim D. S. Chung E. H. Hwang B. K. (2014). Pepper suppressor of the G2 allele of skp1 interacts with the receptor-like cytoplasmic kinase1 and type III effector AvrBsT and promotes the hypersensitive cell death response in a phosphorylation-dependent manner. *Plant Physiol.* 165 76–91. 10.1104/pp.114.238840 24686111 PMC4012606

[B9] Kinoshita E. Kinoshita-Kikuta E. Takiyama K. Koike T. (2006). Phosphate-binding tag, a new tool to visualize phosphorylated proteins. *Mol. Cell. Proteomics* 5 749–757. 10.1074/mcp.T500024-MCP200 16340016

[B10] Li W. Yadeta K. A. Elmore J. M. Coaker G. (2013). The *Pseudomonas syringae* effector HopQ1 promotes bacterial virulence and interacts with tomato 14-3-3 proteins in a phosphorylation-dependent manner. *Plant Physiol.* 161 2062–2074. 10.1104/pp.112.211748 23417089 PMC3613476

[B11] Li X. (2011). Infiltration of *Nicotiana benthamiana* protocol for transient expression via *Agrobacterium*. *Bio Protocol* Bio101:e95.

[B12] Macho A. P. (2016). Subversion of plant cellular functions by bacterial type-III effectors: beyond suppression of immunity. *New Phytol.* 210 51–57. 10.1111/nph.13605 26306858

[B13] Macho A. P. Zipfel C. (2015). Targeting of plant pattern recognition receptor-triggered immunity by bacterial type-III secretion system effectors. *Curr. Opin. Microbiol.* 23C, 14–22. 10.1016/j.mib.2014.10.009 25461568

[B14] Mansfield J. Genin S. Magori S. Citovsky V. Sriariyanum M. Ronald P. (2012). Top 10 plant pathogenic bacteria in molecular plant pathology. *Mol. Plant Pathol.* 13 614–629. 10.1111/j.1364-3703.2012.00804.x 22672649 PMC6638704

[B15] Mukaihara T. Hatanaka T. Nakano M. Oda K. (2016). *Ralstonia solanacearum* type III effector RipAY is a glutathione-degrading enzyme that is activated by plant cytosolic thioredoxins and suppresses plant immunity. *MBio* 7:e00359-16. 10.1128/mBio.00359-16 27073091 PMC4959522

[B16] Mukaihara T. Tamura N. Iwabuchi M. (2010). Genome-wide identification of a large repertoire of *Ralstonia solanacearum* type III effector proteins by a new functional screen. *Mol. Plant Microbe Interact.* 23 251–262. 10.1094/MPMI-23-3-0251 20121447

[B17] Ntoukakis V. Mucyn T. S. Gimenez-Ibanez S. Chapman H. C. Gutierrez J. R. Balmuth A. L. (2009). Host inhibition of a bacterial virulence effector triggers immunity to infection. *Science* 324 784–787. 10.1126/science.1169430 19423826

[B18] Oh M. H. Wang X. Kota U. Goshe M. B. Clouse S. D. Huber S. C. (2009). Tyrosine phosphorylation of the BRI1 receptor kinase emerges as a component of brassinosteroid signaling in *Arabidopsis*. *Proc. Natl. Acad. Sci. U.S.A.* 106 658–663. 10.1073/pnas.0810249106 19124768 PMC2613937

[B19] Peeters N. Carrere S. Anisimova M. Plener L. Cazale A. C. Genin S. (2013). Repertoire, unified nomenclature and evolution of the type III effector gene set in the *Ralstonia solanacearum* species complex. *BMC Genomics* 14:859. 10.1186/1471-2164-14-859 24314259 PMC3878972

[B20] Popa C. M. Tabuchi M. Valls M. (2016). Modification of bacterial effector proteins inside eukaryotic host cells. *Front. Cell. Infect. Microbiol.* 6:73 10.3389/fcimb.2016.00073 PMC4951486 27489796

[B21] Sang Y. Macho A. P. (2017). Analysis of PAMP-triggered ROS burst in plant immunity. *Methods Mol. Biol.* 1578 143–153. 10.1007/978-1-4939-6859-6_11 28220421

[B22] Sang Y. Wang Y. Ni H. Cazale A. C. She Y. M. Peeters N. (2016). The *Ralstonia solanacearum* type III effector RipAY targets plant redox regulators to suppress immune responses. *Mol. Plant Pathol.* 10.1111/mpp.12504 [Epub ahead of print]. 27768829 PMC6638004

