# Supplementary figures and images for: The Ralstonia solanacearum Type III Effector RipAY Is Phosphorylated in Plant Cells to Modulate Its Enzymatic Activity
Source: Front Plant Sci. 2017 Nov 7;8:1899. doi: 10.3389/fpls.2017.01899 (PMC5682030; doi:10.3389/fpls.2017.01899)

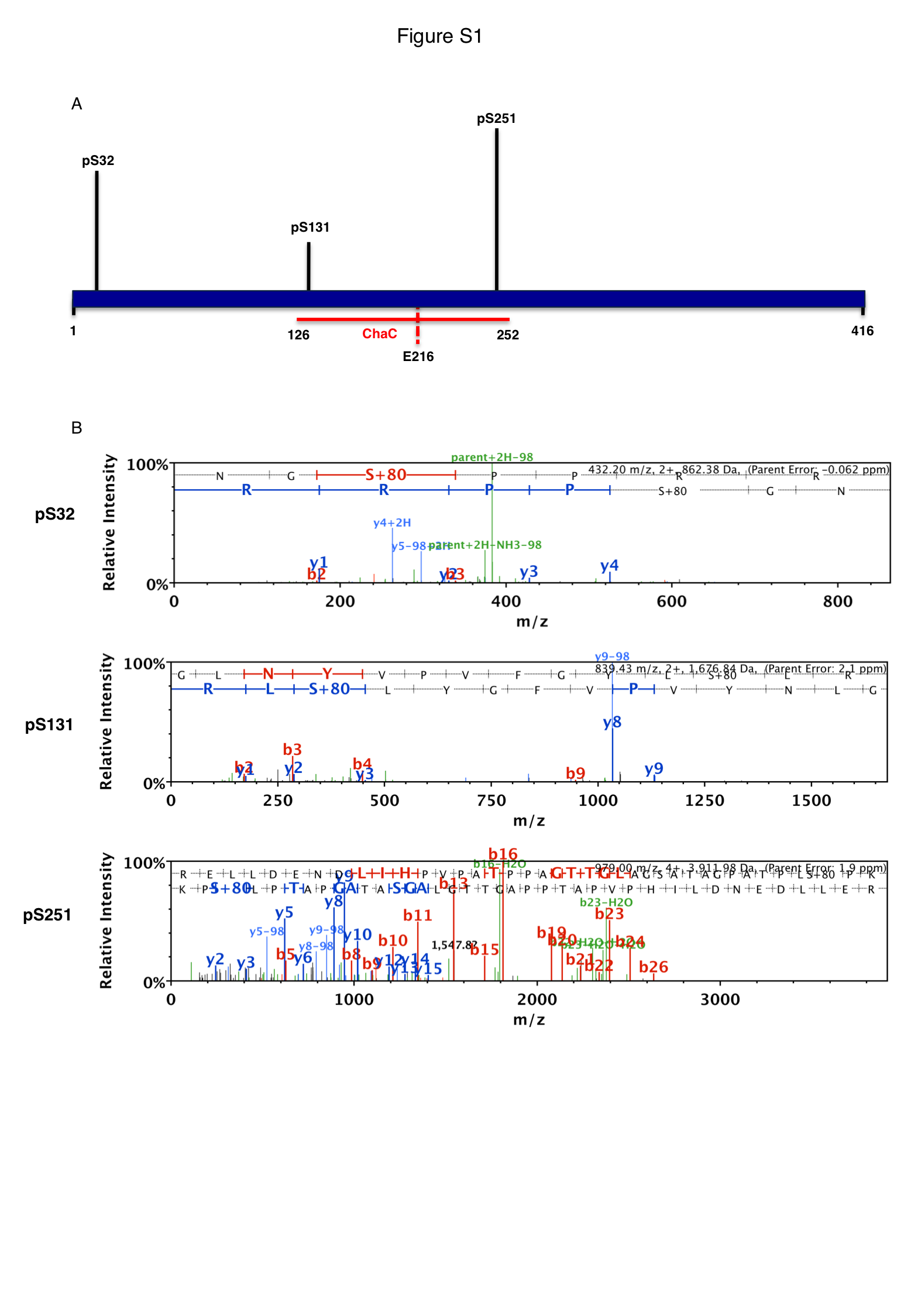

Supplement: FIGURE S1 — RipAY is phosphorylated in several serine residues. (A) Diagram of RipAY indicating the ChaC domain (responsible for the GGCT activity) in red and the observed phosphorylation sites. E216 indicates the catalytic glutamic acid residue essential for GGCT activity. (B) Representative mass spectra of the observed phosphorylated peptides indicated in the Figure 1A. [file Image_1.TIF]

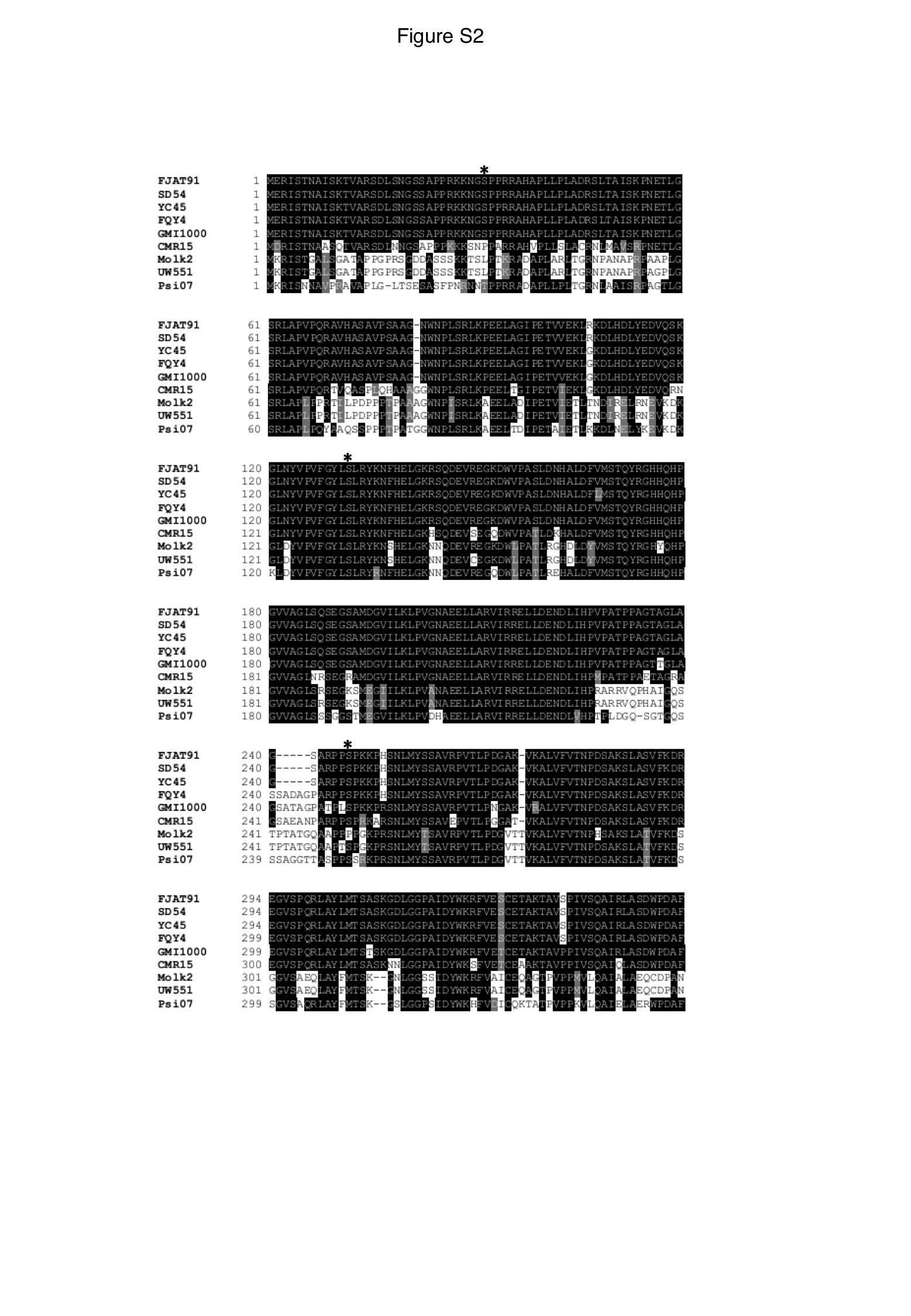

Supplement: FIGURE S2 — Amino acid sequence alignment showing the conservation of the phosphorylated residues among RipAY from different R. solanacearum strains. The strains are representative from the different R. solanacearum phylotypes: phylotype 1: FJAT91, SD54, YC45, FQY4, GMI1000; phylotype 2: UW551 and Molk2; phylotype 3: CMR15; phylotype 4: Psi07. The phosphorylated residues identified in this work in RipAY from GMI1000 are marked with an asterisk [file Image_2.TIF]

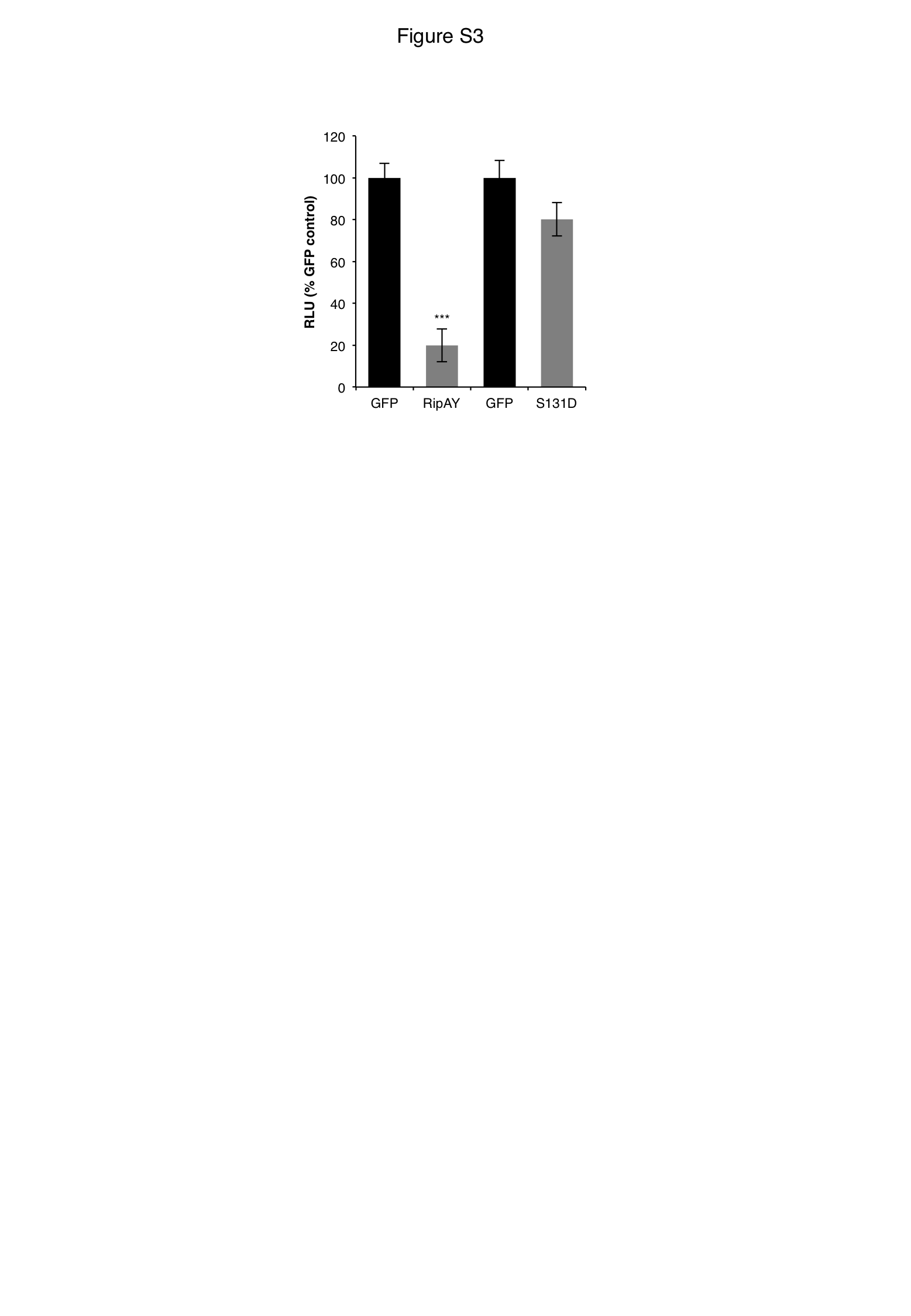

Supplement: FIGURE S3 — The S131D mutation abolishes RipAY suppression of flg22-triggered ROS. A. tumefaciens was used to induce the transient expression of RipAY-GFP (or the S131D mutant) in half of a N. benthamiana leaf and GFP in the other half. The graph shows total oxidative burst triggered by 100 nM flg22 in N. benthamiana tissues 2.5 days post-inoculation (dpi) with A. tumefaciens, and measured in a luminol-based assay as relative luminescence units (RLUs). Values are average ± SE (n = 8). Asterisks indicate significant differences compared to the corresponding GFP control at P < 0.001. The experiments were repeated at least three times with similar results. [file Image_3.TIF]

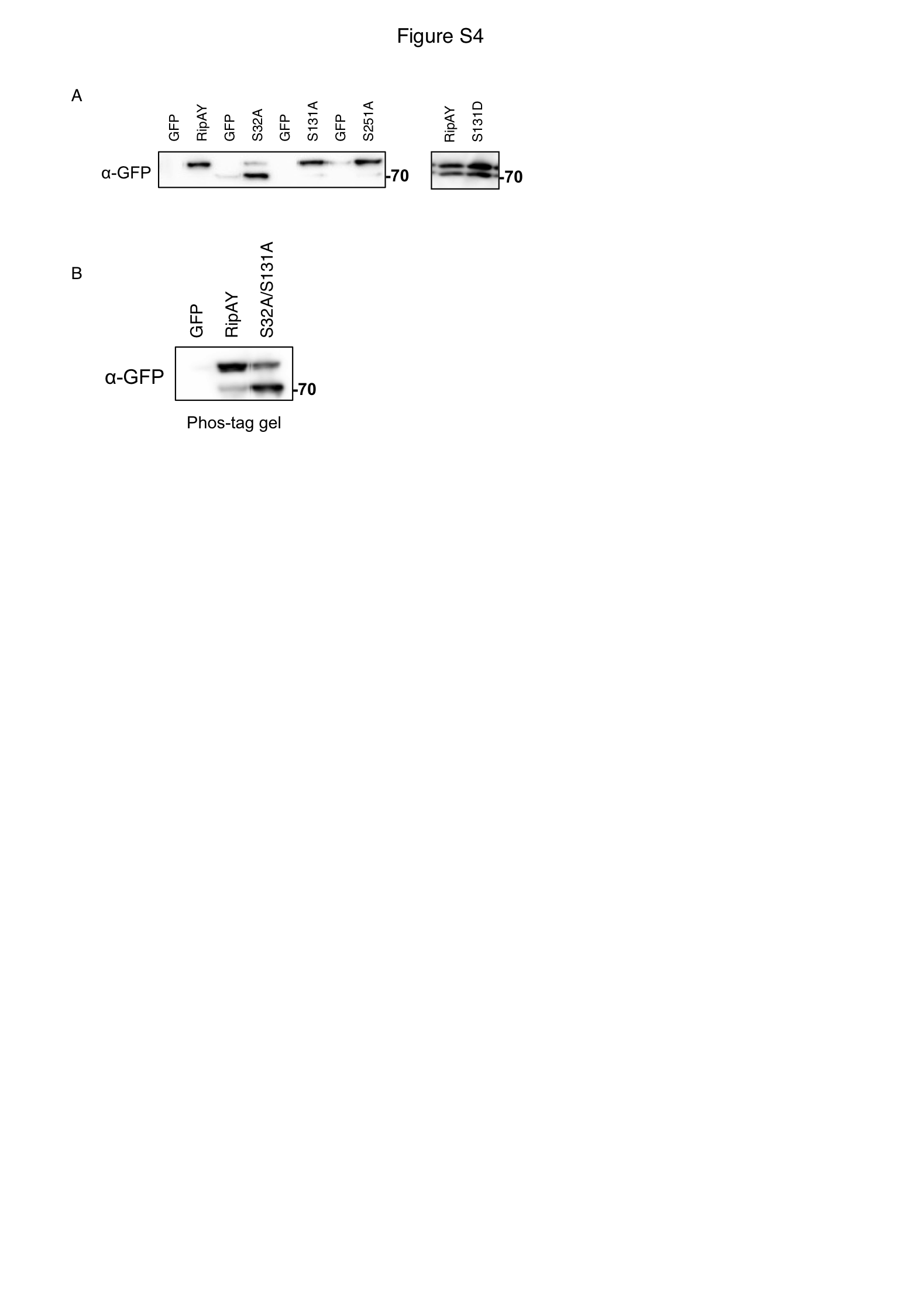

Supplement: FIGURE S4 — All the RipAY mutant variants accumulate to wild-type levels. (A) Western blot assay showing the accumulation of the mutant variants shown in the Figures 1, 2 and Supplementary Figure S3. (B) Western blot assay using a Phos-Tag PAGE gel. Immunoblots were analyzed using anti-GFP antibody. Molecular weight (kDa) marker bands are indicated for reference. [file Image_4.TIF]
