# Supplementary material for: The Ralstonia solanacearum Type III Effector RipAY Is Phosphorylated in Plant Cells to Modulate Its Enzymatic Activity
Source: Front Plant Sci. 2017 Nov 7;8:1899. doi: 10.3389/fpls.2017.01899 (PMC5682030; doi:10.3389/fpls.2017.01899)
Supplement: Supplementary file 5 [file Table_1.DOCX]

Table S1: Primers used for site-directed mutagenesis

| Mutation | Forward primer | Reverse primer |
| --- | --- | --- |
| S32A | gaaagaaaaacggcgcgccgccgagacgc | gcgtctcggcggcgcgccgtttttctttc |
| S131A | ggtgttcggttatctggcgctgcgctacaag | cttgtagcgcagcgccagataaccgaacacc |
| S131D | gtaccggtgttcggttatctggatctgcgctacaagaatttccat | atggaaattcttgtagcgcagatccagataaccgaacaccggtac |
| S251A | cggcaacgccgctcgcccctaaaaaaccg | cggttttttaggggcgagcggcgttgccg |

Letters in red indicate the mutated nucleotides responsible for the amino acid substitution.
